# A 6-year prospective study on work participation and the associated factors in Dutch patients with systemic lupus erythematosus (SLE)

**DOI:** 10.1186/s13075-025-03631-7

**Published:** 2025-09-02

**Authors:** Birgit S. Blomjous, Marieke M. ter Wee, Michel W. P. Tsang-A-Sjoe, Cecile R. L. Boot, Alexandre E. Voskuyl, Irene E. M. Bultink

**Affiliations:** 1 Department of Rheumatology and Clinical Immunology, Amsterdam UMC, Meibergdreef 9, Amsterdam, The Netherlands; 2 Department of Rheumatology and Clinical Immunology, Amsterdam Rheumatology and immunology Center, Amsterdam, The Netherlands; 3 Amsterdam institute for Infection and Immunity, Amsterdam, The Netherlands; 4 Department of Epidemiology and Data Science, Amsterdam UMC, location Vrije Universiteit Amsterdam, De Boelelaan 1117, Amsterdam, The Netherlands; 5 Amsterdam Public Health Research Institute, Societal Participation, Amsterdam, The Netherlands; 6 Department of Public and Occupational Health, Amsterdam UMC, location Vrije Universiteit Amsterdam, Van der Boechorststraat 7, Amsterdam, the Netherlands

**Keywords:** Systemic lupus erythematosus, Employment, Unemployment, Work disability, Work participation, Association, Social support, Job satisfaction, Job retention, Maintaining work

## Abstract

**Background:**

Previous studies showed that many patients with SLE do not have paid work. However, having paid work is important for self-esteem, social contacts and income. It is therefore important to understand the characteristics contributing to work status and in particular identifying modifiable variables to help patients with SLE and their employers to maintain work. The objective of this study is to investigate associations of demographic, disease-related and work characteristics with having and maintaining paid work for ≥ 5 years in patients with SLE over a six-year period.

**Methods:**

All patients diagnosed with SLE, independent of disease duration and under treatment in Amsterdam UMC location VUmc or Reade were invited to participate in the longitudinal Amsterdam SLE cohort (2007–2018). Demographic, disease-related and work characteristics of these patients were analysed. Baseline was defined as the time of study entrance. Generalized Estimating Equations with logit link function were used to identify associations between these characteristics on having paid work. Logistic regression was used to study associations with maintenance of work and work disability.

**Results:**

At baseline, 52% of patients (114/220) were employed, which decreased to 46% (73/157) after six years. The main reported reason for unemployment was because of SLE-related symptoms (63%). Among the 106 patients who were unemployed at baseline, 16% (17/106) gained work during follow-up of whom 47% (8/17) also maintained work. Of the 114 patients employed at baseline, 29% (33/114) remained employed throughout the entire six-year follow-up period. Having paid work over time was associated with younger age, higher level of education, shorter disease duration, lower organ damage and supervisor support. Maintaining employment for ≥ 5 years during follow-up was associated with regular working hours, skill discretion, decision authority and decision latitude. A longer disease duration was associated with work disability at baseline.

**Conclusions:**

This study shows that alongside demographic and disease-related characteristics, also work characteristics are associated with having and maintaining paid work in SLE patients. These characteristics should be taken into consideration when developing interventions to improve sustainable employability in patients with SLE.

## Key messages

1. The unemployment rate in SLE patients is high (54%) and increased over time during follow-up.

2. Maintaining work is associated with more supervisor support, regular working hours, skill discretion, decision authority, decision latitude.

3. Of the unemployed SLE patients at baseline, 16% started working of whom 47% maintained work.

## Background

Systemic lupus erythematosus (SLE) is a chronic systemic auto-immune disease, characterized by alternating periods of active disease and remission. Most patients diagnosed with SLE are of reproductive age [1]. Despite being in the bloom of their lives, these patients are forced by their disease to make adjustments in their lives in terms of work, leisure time activities and family planning [2–5]. These adjustments can substantially impact their quality of life [6–10]. Previous studies showed that many patients with SLE (34–62%) do not have paid work [9, 11–13]. The difficulties of working with SLE consisted of pain, the invisibility of the disease, flares, physical impairments and fatigue [2, 14]. However, having paid work is important for self-esteem, social contacts and income [2, 5]. It is therefore important to understand the characteristics contributing to work status and in particular identifying modifiable variables to help patients with SLE and their employers to maintain work.

Previous studies investigating work outcomes, including having paid work, work disability and productivity loss in patients with SLE, were mostly cross-sectional of design [11, 15–19]. A recently published systematic review including longitudinal studies reporting on possible explanatory characteristics for work outcome in patients with SLE [20], showed that both demographic (such as age, ethnicity, educational level) and disease-related characteristics (including disease duration, disease activity, disease manifestations) were associated with different types of work outcome [11, 15–18, 21–25]. However, only a few studies investigated the role of work characteristics [20, 26]. The studied work characteristics encompassed physical and cognitive job demands, type of occupation, type of industry, years since regular work, occupational prestige, job demands, and job control [22, 24, 27, 28]. Interestingly, recently published literature showed that also work characteristics such as job satisfaction and support at the workplace may facilitate the maintenance of paid work [29–33]. However, these important work characteristics were not included in the previously mentioned studies.

The aim of this study is therefore twofold: (1) to prospectively study the employment rate and transitions in work status over six years of patients with SLE, and (2) to study which demographic, disease-related and work characteristics (including job satisfaction and social support) are associated with having paid work, maintaining work and work disability. Knowledge on this topic is needed to enable the development of strategies to improve sustainable employment in patients with SLE.

## Methods

### Study population

All patients diagnosed with SLE, independent of disease duration, fulfilling the 1997 update of the revised American College of Rheumatology (ACR) criteria, aged ≥ 18 years and under treatment in Amsterdam UMC location VU University medical center (VUmc) (a tertiary center) and Reade (a secondary care facility), were invited by their treating rheumatologist to participate in the prevalent longitudinal Amsterdam SLE cohort [34]. Data from all patients included between October 2007 and March 2018 in this Dutch observational prospective cohort were used for this study [34, 35]. This total populationhas been followed over time. Patients with missing work status data at baseline and those without follow-up visits were excluded from all analyses.

Patients were enrolled in this cohort study and underwent five follow-up assessments with yearly intervals, covering a total of six years follow-up. The first visit during inclusion in the cohort was determined as the baseline. During baseline and yearly follow-up visits, all patients were investigated by a study physician. Questionnaires were used to collect data on demographic and work characteristics. For this study, only the data of the 220 patients with a baseline visit and at least one follow-up visit were taken into account. Not all patients were followed during six years.

The study protocol was approved by the Medical Ethics Committee of Amsterdam UMC, location VUmc (study number: NL17200.029.07). The study was conducted in accordance with the Declaration of Helsinki/Good Clinical Practice. All patients provided written informed consent.

### Variables

#### Outcome measures

The primary outcome measure was having paid work, as reported by the patient, regardless of contract hours (‘Do you currently perform paid work in employment or as a self-employed person?’ Answer options: Yes/No). Secondary outcome measures were demographic, disease-related and work characteristics associated with paid work, maintenance of paid work during at least five years of follow-up and work disability. Work disability was defined as temporary or permanent work disability as reported by the patient. In the Netherlands, workers are entitled for work disability benefits after two years of sick leave. The employer is responsible for continuing salary payments during the first two years of sick leave. After two years, when full return to work is not possible, the worker is entitled to work disability benefits. The amount of work disability benefits is established following an assessment by a social insurance physician and based on loss of earning capacity from the previous job.

Patients with a work disability were questioned about the reason for their work disability (SLE-related or other), the type of work disability (partial/complete) and whether they were currently receiving a disability pension for SLE (yes/no).

The independent variables under investigation were categorized as demographic, disease-related, and work characteristics.

#### Demographic characteristics

Collected demographic data comprised gender, age in years, ethnicity (Caucasian versus non-Caucasian), marital status (single versus living together), and duration of education in years (as deduced from the specified highest attained educational level).

#### Disease-related characteristics

At baseline, disease-related characteristics comprised the year of SLE diagnosis, disease duration (defined as the time from diagnosis until inclusion), medication usage (including non-steroidal anti-inflammatory drugs (NSAIDs), prednisone, conventional synthetic disease-modifying anti-rheumatic drugs (csDMARDs), and biological DMARDs (bDMARDs)), the Systemic Lupus Erythematosus Disease Activity Index 2000 (SLEDAI-2 K) score [36], and the organ damage index measured using the Systemic Lupus International Collaborative Clinics/American College of Rheumatology Damage Index (SDI) [37]. The median disease duration was used to categorize patients in those with short versus long disease duration. During each follow-up visit, medication usage, self-reported disease activity, SLEDAI-2 K, SDI, and the occurrence of any flare in the past year according to the SELENA-SLEDAI flare criteria (yes/no) [38] were assessed. Data on disease-related variables, flares [38], medication, and the SDI [37] were obtained from the patient medical records.

#### Work characteristics

At baseline, patients were asked about prior paid work (yes/no), paid work at the time of diagnosis (yes/no). Additionally, if they had stopped working, they were asked to specify the reason for discontinuation. Patients with paid work were further questioned about the type of work (blue collar (physical work) versus white collar (typically more office work)), employment contract status versus self-employment, weekly working hours (full-time paid work defined as ≥ 36 h per week), participation in working in evening/night shifts (yes/no), involvement in irregular working hours (yes/no), and the availability of supervisor support to adjust working conditions (no help (needed) versus adjustments made at work, with specifications of the adjustments). Each year, patients were asked if their work status changed compared to the previous year (no/yes: what has changed? ). Patients with paid work were also asked if they were dissatisfied with their work situation (yes/no), experienced workload (normal, too high, too low), and if they preferred to work fewer or more hours (yes/no). The validated Dutch version of the Job Content Questionnaire (JCQ) was assessed to explore job demands, control, and support [39]. Patients rated 28 items on a four-point ordinal Likert scale (1 = totally disagree, 2 = disagree, 3 = agree, 4 = totally agree). Using these items, the predefined subscales by Karasek (skill discretion, decision authority, decision latitude - a combination of the two previously mentioned subscales, psychological job demands, coworker support, and supervisor support) were constructed using the JCQ user’s guide [39, 40]. The JCQ was introduced in 2013, after the study’s initiation, thus not all patients responded to it from the baseline visit of the study. If patients indicated they were not having paid work at follow-up, no additional JCQ questions were asked.

### Statistical analysis

Descriptive statistics are presented as percentages for categorical variables, means with standard deviations (SD) for continuous variables, and median with interquartile ranges (IQR) for skewed data.

To address missing data on work status, four three assumptions were applied: (1) Patients who initially declared not having paid work at baseline but nevertheless completed all questions concerning paid work were considered as having paid work. This is because patients without paid work would not logically be able to respond to these job-specific questions, indicating a likely discrepancy in their baseline work status declaration; (2) Patients on sick leave were categorized as having paid work since this best suited real-world practice; (3) Patients who answered the JCQ or the question on workload were assumed to have paid work and in case the work situation of patients contained inconsistencies and they did not answer the JCQ or the question on workload, these patients were assumed not to have paid work, as these questions could only be completed by patients having paid work. Assumptions were only made if the patient was not lost to follow-up during the six years of follow-up.

To investigate the primary aim, the employment rate and transitions in work status over six years, descriptive statistics were used. For the secondary aim, we used generalized estimating equations (GEE) with a logit link function to investigate longitudinal data and to account for repeated measures within individuals over time [41, 42]. All participants with at least one follow-up assessment contributed to the analysis; complete follow-up across all six years was not required. An exchangeable correlation structure was specified for simplicity and due to the limited number of repeated observations per participant. Data were assumed to be missing completely at random (MCAR), consistent with the assumptions underlying GEE. Missing responses to the question on change in work situation were conservatively imputed as ‘not having work’, representing a worst-case scenario approach. Furthermore, imputations were made based on responses to the JCQ and workload questions; for example, if a patient initially reported no paid work at year 2 but subsequently completed job-specific questionnaires in year 3 indicating unchanged work status, their year 3 work status was updated to ‘having paid work’. GEE was used for fixed variables such as demographic characteristics, disease duration at baseline and the variable flare occurrence in the past year. A time-lag GEE model, which relates characteristics to the subsequent year’s outcome of having paid work (yes/no), was used for non-fixed characteristics. No time-lag model was used for flare rate, since data on flare rate in the past year were only available from yearly assessments at time-points one year until five years after baseline but no data on flare occurrence in the year before inclusion in the study were available. Associations with work characteristics were analyzed in patients who stated having work at baseline and were aged ≤ 65 years throughout follow-up. The other characteristics were studied in the total population (working and non-working patients).

To study firstly which demographic, disease-related and work characteristics at baseline were associated with maintaining work for ≥ 5 years, and to study secondly which demographic and disease-related characteristics at baseline were associated with work disability in patients without work at baseline, cross-sectional analyses were performed by using logistic regression analyses.

Results are presented as odds ratios (OR) with 95% confidence intervals (CI) and *p*-values. All analyses were carried out using SPSS Statistics V28 (IBM Corp., Armonk, NY, USA).

## Results

### Study population

A total of 261 patients were included in the Amsterdam SLE cohort and 220 patients fulfilled the inclusion criteria for this study. Forty-one patients were excluded because of missing baseline or follow-up data on having paid work). These excluded patients were of similar age (mean 42 years (SD 16)), had a low median SLEDAI-2 K (median 4; IQR [1–10]) and SDI (median 0; IQR [0–2]), and comparable work status.

### Demographic, disease-related, and work characteristics at baseline in the total study population

Table 1 presents the demographic, disease-related, and work characteristics of the 220 included patients at baseline. About half of thepatients were employed at baseline, of whom nine were self-employed and six were on sick leave. Four patients on sick leave indicated that they were reintegrating into work through occupational therapy. From the patients without paid work at baseline, 94% had prior work experience, and the majority ceased working due to SLE-related symptoms (63%).

**Table 1:** Baseline demographic, disease related and work characteristics

	Total (*n* = 220)	Employed (*n* = 114)	Unemployed (*n* = 106)
**Demographic characteristics**			
Female	199 (90.5%)	104 (91.2%)	95 (89.6%)
Caucasian ethnicity	153 (69.5%)	80 (70.2%)	73 (68.9%)
Age (years), mean (SD)	41 (12.5)	36 (8.9)	47 (13.6)
Married or living together [1]	135 (63.4%)	71 (64.0%)	64 (62.7%)
Number of children [2]	1 [0–2]	0 [0–2]	1 [0–2]
Years of education [3]	16 [12–17]	16 [13–17]	12 [11–16]
**Disease related characteristics**			
Disease duration in years	5 [1–11]	4 [1–8]	7 [2–12]
Nephritis in history	53 (24.1%)	23 (20.2%)	30 (28.3%)
Arthritis at baseline	142 (64.5%)	72 (63.2%)	70 (66.0%)
SDI	1 [0–2]	0 [0–1]	1 [0–3]
SLEDAI-2K score [4]	4 [2–6]	4 [2–6]	2 [2–6]
Medication			
• NSAID	66 (30.0%)	34 (29.8%)	32 (30.2%)
• Prednisone	116 (52.7%)	52 (45.6%)	64 (60.4%)
• csDMARD	192 (87.3%)	101 (88.6%)	91 (85.8%)
• bDMARD	3 (1.4%)	2 (1.8%)	1 (0.9%)
**Work status**			
Having work in the past [6]			98 (94.2%)
Having work at diagnosis [7]			48 (47.1%)
Receiving disability pension due to SLE [8]			57 (58.2%)
Change of job (partly) due to SLE [9]		14 (48.2%)	
**Work characteristics**			
Reported reason to stop working [10]			
• SLE-related symptoms			58 (62.8%)
• Retirement			4 (4.3%)
• Employment contract not extended			4 (4.3%)
• Other			27 (28.7%)
Having an employment contract [11]		102 (91.9%)	
White collar job [12]		67 (61.5%)	
Hours of work per week, mean (SD) [13]		27 (11.5)	
Working fulltime^14 #^		34 (30.9%)	
Having evening and night shifts [15]		24 (21.6%)	
Having irregular working hours [16]		27 (24.1%)	
Received no help or did not need help of employer in last 12 months to improve working conditions (of patients with employment contract) [17]		53 (52.0%)	
Experienced workload [18]			
• Normal		79 (72.5%)	
• Too high		28 (25.7%)	
• Too low		2 (1.8%)	
Dissatisfied with work situation [19]		23 (21.3%)	
Would like to work less [20]		35 (32.4%)	
Would like to work more [21]		8 (20.0%)	
Job Content Questionnaire^^^			
• Skill discretion [22]		36.6 (6.0)	
• Decision authority [23]		34.2 (8.6)	
• Decision latitude [24]		71.6 (12.6)	
• Psychological job demands [25]		32.6 (5.1)	
• Supervisor support [26]		11.4 (2.5)	
• Coworker support [27]		13.3 (1.8)	
Working during follow-up [28]			
• 1–4 years		52 (45.6%)	15 (88.2%)
• ≥ 5 years^*^		62 (54.4%)	2 (11.8%)

Legend: Baseline demographic, disease related and work characteristics of the total population (*n* = 220), employed patients (*n* = 114) and unemployed patients (*n* = 106). Data are presented as n (%), mean (SD) or median [interquartile range]

(1) Employed: *n* = 111, Unemployed: *n* = 102. (2) Employed: *n* = 111, Unemployed: *n* = 102. (3) Employed: *n* = 113, Unemployed: *n* = 104. (4) Employed: *n* = 114, Unemployed: *n* = 105. (5) Employed: *n* = 112, Unemployed: *n* = 103. (6) Unemployed: *n* = 104. (7) Unemployed: *n* = 102. (8) Unemployed: *n* = 98. (9) Employed: *n* = 29/109 change of job. (10) Unemployed: *n* = 93. (11) Employed: *n* = 111. (12) Employed: *n* = 109. (13) Employed: *n* = 108. (14) Employed: *n* = 110. (15) Employed: *n* = 111. (16) Employed: *n* = 112. (17) Employed: *n* = 102. (18) Employed: *n* = 109. (19) Employed: *n* = 108. (20) Employed: *n* = 108. (21) Employed: *n* = 40. (22) Employed: *n* = 25. (23) Employed: *n* = 26. (24) Employed: *n* = 25. (25) Employed: *n* = 25. (26) Employed: *n* = 22. (27) Employed: *n* = 24. (28) Employed: *n* = 114, Unemployed: *n* = 17

^#^ Fulltime work = 36 h of work per week or more

^^^ A higher score on for example supervisor support, means more supervisor support

^*^ In total, 64 patients were working during ≥ 5 years. At baseline, 2 patients were not working. They started working in the year thereafter and therefore 62 patients are mentioned in this table

NSAID = Non Steroidal Anti Inflammatory Drug; csDMARD = conventional synthetic Disease Modifying AntiRheumatic Drug (azathioprine, ciclosporin A, cyclophosphamide, (hydroxy)chloroquine, leflunomide, methotrexate, mycophenolate mofetil, tacrolimus); bDMARD = biological Disease Modifying AntiRheumatic Drug (anti-B cell therapy, anti-T cell therapy, interleukin inhibitors, TNF alpha inhibitors); SDI = Systemic Lupus International Collaborative Clinics/American College of Rheumatology Damage Index; SLEDAI = Systemic Lupus Erythematosus Disease Activity Index 2000

### Transitions in work status in the total population

**Fig. 1:**
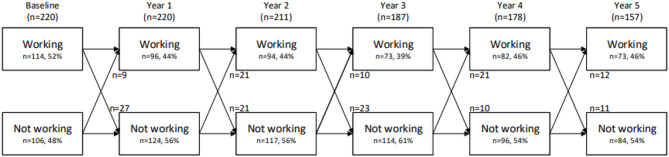
Transitions in work status of patients with SLE. Shows the transitions patients are making between having paid work and not having paid work. In total, 165 transitions were made by 82 patients. Twenty-five patients made only one transition (*n* = 6 start working and *n* = 19 stop working). Attrition: The sample size decreased over time due to missing data on work status. In year 2, 2 patients did not yet have data on the second year after inclusion and 7 patients were lost to follow-up. In year 3, 1 patient died, 5 patients did not yet have data of the third year after inclusion, and 27 patients were lost to follow-up. In year 4, 1 patient died, 9 patients did not yet have data on the fourth year after inclusion and 32 patients were lost to follow-up. In year 5, 3 patients died, 17 patients did not yet have data on the fifth year after inclusion and 43 patients were lost to follow-up. Imputations: • In approximately 4% of visits, transitions in employment were imputed due to the answers of the JCQ and/or work load. • In 12% of visits a missing answer on a change in work situation was imputed as ‘not having work’ (worst case scenario). In 11% of cases no follow-up data was available and this was not imputed but regarded as lost to follow-up

Figure 1 presents transitions in working status over six-year follow-up. The percentage of employed patients decreased from 52% (*n* = 114) at baseline to 46% (*n* = 73) after six years. Only one patient stopped working due to retirement. Among the 114 patients with paid work at baseline, 58% (*n* = 66) experienced intermittent work cessation during follow-up. Of these, 14 patients (*n* = 21%) remained unemployed and six (9%) stopped working at their last visit. The remaining patients showed transitional changes between work statuses. In total, 77 patients stopped working one or more times during follow-up. These patients were often female (*n* = 68, 88%) with a mean age of 37 years (SD 9.2 years) and a median disease duration of 3 years [IQR 1–9 years]. In the year preceding the loss of work, dissatisfaction with the work situation was reported by 39% (*n* = 29/75) and 30% (*n* = 22/73) assessed that their workload was too high.

**Fig. 2:**
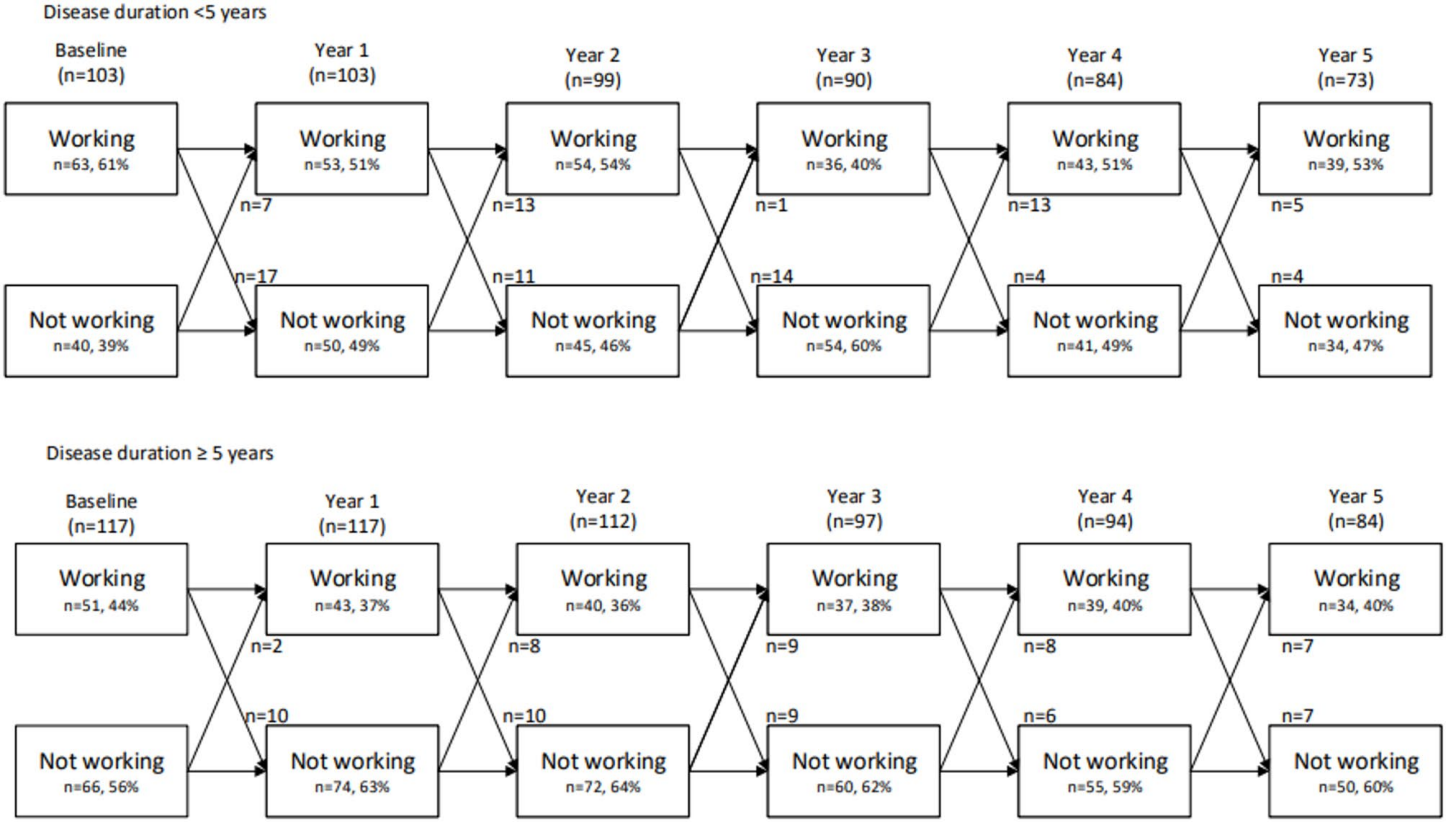
Transitions in work status according to disease duration. shows transitions in work status according to disease duration (< 5 years or ≥ 5 years)

Of the 106 patients without work at baseline, 17 patients (16%) started working at least once during follow-up, with eight patients (47%) maintaining paid work until their last follow-up measurement. These 17 patients had a lower mean age (32 versus 50 years), had a shorter median disease duration (3 versus 7 years) and longer median years of education (16 versus 12 years) compared to unemployed patients not restarting working. Figure 2 presents the transitions in work status based on disease duration. Patients with a disease duration of < 5 years at baseline had an odds ratio (OR) of 0.55 (95% CI [0.354; 0.865], *p* = 0.009) for having paid work over six years compared to patients with a disease duration ≥ 5 years. Conversely, among the 114 patients having paid work at baseline, 56% (*n* = 64) maintained work for at least five years, of whom 38% (*n* = 43/114) retained work in five consecutive years (Supplementary Figure S1). The remaining 21 patients did not work during one year of the follow-up period, but worked during all other years. Only 33 patients (29%) were employed at all visits (Supplementary Figure S1).

### Longitudinal associations with having paid work

In Tables 2 and 3 the results of the univariable longitudinal GEE analyses of demographic, disease-related, and work characteristics associated with having paid work (yes versus no) are shown. Demographic and disease-related characteristics were analyzed for all patients (*n* = 220), while work characteristics were only analyzed in working patients ≤ 65 years throughout the study period (*n* = 113). Having paid work over time was significantly associated with younger age, higher educational level, shorter disease duration, and lower SDI. Out of the work characteristics analyzed, only having supervisor support was significantly associated with having paid work over time. A lower degree of supervisor support (OR 0.92, 95% CI [0.842; 0.997], *p* = 0.043) and a lower degree of coworker support (OR 0.88, 95% CI [0.780; 0.995], *p* = 0.041) were significantly linked to work cessation the following year.

**Table 2:** Univariable GEE analyses of demographic and disease related characteristics with having paid work compared to not having paid work as outcome measure

	OR	[95% CI]	*p*-value
**Demographic characteristics**			
Gender	0.99	0.473; 2.069	0.977
Age	0.92	0.903; 0.941	**< 0.001**
Education	1.23	1.137; 1.322	**< 0.001**
Marital status	0.99	0.618; 1.589	0.969
**Disease related characteristics**			
Disease duration	0.95	0.925; 0.984	**0.003**
Development of nephritis	0.87	0.584; 1.294	0.490
Development of arthritis	1.25	0.966; 1.608	0.091
SDI	0.71	0.619; 0.813	**< 0.001**
SLEDAI-2K	0.99	0.968; 1.021	0.677
Development of ≥ 1 major flare in previous year			
• Year 1	2.02	0.746; 5.468	0.166
• Year 2	0.82	0.351; 1.911	0.645
• Year 3	1.94	0.606; 6.179	0.265
• Year 4	0.92	0.364; 2.324	0.860
• Year 5	1.29	0.427; 3.892	0.652

Univariable (longitudinal) GEE analyses of demographic and disease related characteristics in the total patient population with having paid work compared to not having paid work as outcome measure (*n* = 220 × 5 follow-up measurements).

CI = confidence interval; OR = odds ratio; SDI = Systemic Lupus International Collaborative Clinics/American College of Rheumatology Damage Index; SLEDAI = Systemic Lupus Erythematosus Disease Activity Index 2000

**Table 3:** Univariable GEE analyses of work characteristics in working patients at baseline with having paid work compared to not having paid work during follow-up as outcome measure

	OR	[95% CI]	*p*-value
**Work characteristics**			
White/blue collar job	0.80	0.436; 1.462	0.465
Evening and night shifts	0.67	0.344; 1.290	0.228
Irregular work	0.55	0.288; 1.029	0.061
Experienced workload	0.89	0.526; 1.506	0.665
Dissatisfied with work situation	1.08	0.665; 1.747	0.760
Would like to work less	0.94	0.575; 1.545	0.815
Would like to work more	0.68	0.370; 1.245	0.210
Job Content Questionnaire			
• Skill discretion	1.00	0.988; 1.018	0.695
• Decision authority	1.00	0.987; 1.016	0.859
• Decision latitude	1.00	0.994; 1.008	0.784
• Psychological job demands	1.04	0.991; 1.087	0.117
• Supervisor support	1.11	1.017; 1.221	**0.021**
• Coworker support	1.12	0.983; 1.276	0.089

Univariable (longitudinal) GEE analyses of work characteristics in working patients at baseline and aged ≤ 65 years throughout follow-up (*n* = 113 × 5 follow-up measurements) with having paid work compared to not having paid work during follow-up as outcome measure. Of the 114 working patients at baseline, one patient reached retirement age during follow-up

CI = confidence interval; OR = odds ratio

### Cross-sectional associations with maintenance of paid work and work disability

The cross-sectional analyses between demographic, disease-related, and work characteristics and the maintenance of paid work and work disability are shown in Tables 4 and 5, respectively. For patients with paid work at baseline, none of the demographic and disease-related characteristics were associated with maintaining paid work for ≥ 5 years. However, maintaining paid work was associated with work-related factors such ashaving regular working hours, skill discretion, decision authority, and decision latitude. Of interest, no association was found between maintaining paid work and supervisor support.

**Table 4:** Univariable logistic regression analyses of demographic, disease related and work characteristics with maintenance of work for ≥ 5 years as outcome measure

	OR	[95% CI]	*p*-value
**Demographic characteristics**			
Gender	0.83	0.225; 3.021	0.771
Age	0.98	0.938; 1.020	0.292
Education	1.07	0.917; 1.238	0.410
Marital status	1.37	0.627; 2.977	0.432
**Disease related characteristics**			
Disease duration	0.99	0.938; 1.037	0.594
Nephritis in history or at baseline	1.11	0.443; 2.801	0.818
Arthritis in history or at baseline	1.14	0.530; 2.438	0.743
SDI	0.97	0.660; 1.416	0.861
SLEDAI-2K score	1.03	0.935; 1.139	0.531
**Work characteristics**			
White/blue collar job	0.65	0.300; 1.418	0.281
Evening and night shifts	0.50	0.202; 1.261	0.143
Irregular work	0.39	0.160; 0.958	**0.040**
Experienced workload	1.18	0.546; 2.563	0.670
Dissatisfied with work situation	1.32	0.515; 3.376	0.564
Would like to work less	1.38	0.610; 3.129	0.438
Would like to work more	1.29	0.272; 6.069	0.751
Job Content Questionnaire			
• Skill discretion	0.97	0.945; 0.995	**0.018**
• Decision authority	0.97	0.943; 0.994	**0.017**
• Decision latitude	0.98	0.972; 0.997	**0.018**
• Psychological job demands	1.00	0.849; 1.187	0.964
• Supervisor support	0.88	0.610; 1.260	0.477
• Coworker support	0.76	0.431; 1.340	0.343

Univariable (cross-sectional) logistic regression of demographic, disease related and work characteristics of patients having paid work at baseline and maintaining work during at least five years (*n* = 64) as outcome measure

CI = confidence interval; OR = odds ratio; SDI = Systemic Lupus International Collaborative Clinics/American College of Rheumatology Damage Index; SLEDAI = Systemic Lupus Erythematosus Disease Activity Index 2000

**Table 5:** Univariable analyses of demographic and disease related characteristics with work disability as outcome measure

	OR	[95% CI]	*p*-value
**Demographic characteristics**			
Gender	1.11	0.304; 4.084	0.871
Age	0.99	0.962; 1.021	0.567
Education	1.03	0.932; 1.133	0.590
Marital status	0.56	0.228; 1.354	0.196
**Disease related characteristics**			
Disease duration	1.08	1.019; 1.149	**0.010**
Nephritis in history or at baseline	2.13	0.836; 5.430	0.113
Arthritis in history or at baseline	1.09	0.472; 2.531	0.835
SDI	1.18	0.943; 1.475	0.147
SLEDAI-2 K score	1.00	0.914; 1.096	0.986

Univariable (cross-sectional) logistic regression analysis of demographic and disease related characteristics of patients having work disability at baseline (*n* = 62) as outcome measure

CI = confidence interval; OR = odds ratio; SDI = Systemic Lupus International Collaborative Clinics/American College of Rheumatology Damage Index; SLEDAI = Systemic Lupus Erythematosus Disease Activity Index 2000

Among patients without paid work at baseline, work disability at baseline was only associated with longer disease duration, although the effect size was small.

## Discussion

The primary aim of this study was to investigate the employment rate of patients with SLE and transitions in employment rates during follow-up. This study revealed an initial employment rate of 54% among patients aged ≤ 65 years. The current findings regarding employment rates align with the ranges (37–68%) reported by other studies involving patients with SLE [9, 11–14, 43–45]. The employment rates in our cohort confirm that work participation among patients with SLE is lower compared to the general Dutch population, where 66–73% of individuals aged 15–75 years were employed from 2014 onwards [9, 46]. In 2016, 82% of Dutch women aged 40–44 years (the mean age in this cohort study was 41 years) were employed [47]. Over the six-year follow-up period, the employment rate decreased from 52 to 46%. The decrease in employment rate during follow-up can not be attributed to retirement since only one of the employed patients reached retirement age during follow-up. The decrease in employment rate of SLE patients during the disease course found in our study is confirmed by two other studies. A longitudinal study in German patients with SLE demonstrated an employment rate of 57% at inclusion and decreasing to 48% four years later [45]. A study by Yelin et al. reported a decrease of patients working at the time of diagnosis (74%) to an average of 54% twelve years later [22]. The cessation of work by many patients may be influenced by the challenges of harmonising disease symptoms and organ manifestations with work [25, 26]. Possibly, many patients are able to maintain work at the onset of disease (and may be on sick leave for a while), but finally fail to maintain work. Fatigue, pain, the invisibility and fluctuating nature of SLE are described as difficulties in maintaining work [14]. This underlines the seriousness and impact of the diagnosis SLE on patients.

Among all patients, 77 patients stopped working one or more times during the follow-up period. Patients transitioning between work statuses, often transition multiple times, suggesting temporary changes in work status. Among the 106 patients who were unemployed at baseline, 16% started working (*n* = 17), and eight of them were able to maintain work until their last follow-up visit. This finding illustrates difficulties in maintaining paid work among patients with SLE, which is confirmed in the literature [10].

In the current study, having paid work was significantly associated with younger age, a higher level of education, shorter disease duration, and lower SDI. These findings are similar to differences between workers and non-workers in the total Dutch population, in which labour market participation is highest among young people with a high level of education [48]. Furthermore, having paid work in patients with SLE was significantly associated with a shorter disease duration and a lower SDI. Both demographic and disease-related characteristics associated with paid work align with findings in the literature, as summarized in a recently published systematic review on work participation in patients with SLE [20]. For example, being higher educated was associated with favorable work outcome in other studied SLE populations [17, 18, 22], a pattern also observed in studies involving patients with other rheumatic diseases [49–51].

Our study is one of the few studies also investigating longitudinal associations between work characteristics and work outcome [22, 24, 25, 27, 28]. In contrast to the findings of the present study, Yelin et al. showed that the duration until work loss in employed SLE patients was associated with amongst others higher psychological job demands [22], and they identified a lower risk of work loss in employed patients with higher psychological job demands [24]. While psychological job demands did not show an association with the presence of maintenance of paid work in our study, this discrepancy might be attributed to a presence of ‘positive stress’ within our patient population. Remarkably, our population showed higher psychological job demands and higher decision latitude compared to the reference Dutch group used by Karasek [39].

Maintenance of paid work for ≥ 5 years occurred in only 29% of patients and was associated with having regular working hours, skill discretion, decision authority and decision latitude. Regular, plannable work with need for skills, with the authority to make decisions and having control over tasks, and participation in organisational matters could make a job interesting and challenging [39]. Work satisfaction is another important characteristic for maintaining work [3, 14]. Among patients leaving work for the first time, dissatisfaction with their work situation was reported in 39% of cases in the year preceding the cessation of work. While work satisfaction is very important to maintain work, it was not significantly associated with work outcomes in this study. However, the small sample size might contribute to these findings.

One of the strengths of this study is the longitudinal analysis of work status in patients with SLE, allowing the investigation of characteristics and their associations with subsequent work status. Furthermore, this study is the first longitudinal study assessing social support at work in SLE patients. Limitations include the lack of a yearly work status follow-up question, necessitating assumptions based on responses to the JCQ and workload question. In addition, reasons for work status transitions were not sufficiently available due to missing data. A key limitation of our longitudinal analysis is the handling of this missing data. While participants were included if they had data for at least one follow-up point, GEE assumes that data are missing completely at random (MCAR), which may not hold in practice. For example, participants who dropped out of the study may have had different work outcomes than those who remained. Sensitivity analyses have not been performed to assess the robustness of the handling of missing work status data, this could be investigated in follow-up research. Additionally, we used an exchangeable correlation structure, which assumes constant correlation between time points. This may not adequately reflect temporal trends, such as a progressive increase in risk of work disability or unemployment. These modeling decisions were made to balance analytical rigor with the constraints of our sample size and follow-up structure, but we acknowledge that they may limit the precision and generalizability of our findings. Furthermore, we were limited in our ability to conduct multivariable regression analyses. The low number of events restricted our capacity to adjust for potential confounders or explore effect modification without risking model overfitting or producing unstable estimates. Another limitation included reasons for loss to follow-up were not systematically documented. Furthermore, no time-lag model for flare rate could be used, limiting causal interpretations. An unexpected finding was that higher scores on certain JCQ work characteristics, such as skill discretion, decision authority, and latitude, were associated with lower odds of maintaining work over ≥ 5 years. While these measures generally reflect greater autonomy and control at work, which are often considered beneficial, it is possible that higher work demands or responsibilities captured by these scales may also contribute to increased stress or strain, negatively affecting work sustainability. Additionally, residual confounding or unmeasured factors may influence these associations. Therefore, these results should be interpreted cautiously, recognizing the complex and potentially bidirectional relationships between work characteristics and long-term employment outcomes.

The results of our study demonstrate that regular working hours should be encouraged to support maintaining paid work over time in patients with SLE. In addition, based on our findings, future intervention studies to improve sustainable employability in patients with SLE should include research on support of autonomy (skill discretion, decision authority, decision latitude) and supervisor support. Likely, autonomy and supervisor support will help all workers in general. Therefore, generalised policy changes encouraging these types of support are needed since this will have a broader base of support and will include workers with SLE. To provide tailored suggestions specific for patients with SLE, validated characterisation of work characteristics might help in future studies. Furthermore, future qualitative research is needed to provide more in depth insight in reasons and circumstances of patients’ employment situation related to these identified factors. Such research would support the development and implementation of evidence based interventions to support patients with SLE.

## Conclusions

The results of this study show the employment rate in patients with SLE is relatively low and decreases over time. Despite improvements in treatment over the last decades, patients with SLE still face a high unemployment rate with substantial life impacts. Of the unemployed patients, 16% start working but only 47% are able to sustain work. Demographic, disease-related and work characteristics influence work outcomes. Having work is associated with supervisor support, while maintaining work is associated with regularity, skill discretion, decision authority, and decision latitude. Low supervisor and coworker support is associated with leaving work the following year. Intervention strategies should be developed considering these work characteristics to help SLE patients maintain employment.

## Data Availability

The datasets used and/or analysed during the current study are available from the corresponding author on reasonable request.

## Abbreviations

ACR: American College of Rheumatology
bDMARD: biological Disease-Modifying Anti-Rheumatic Drugs
CI: Confidence Interval
csDMARD: conventional synthetic Disease-Modifying Anti-Rheumatic Drugs
GEE: Generalized Estimating Equations
JCQ: Job Content Questionnaire
NSAID: Non-Steroidal Anti-Inflammatory Drugs
OR: Odds Ratio
SD: Standard Deviation
SDI: Systemic lupus international collaborative clinics/american college of rheumatology Damage Index
SLE: Systemic Lupus Erythematosus
SLEDAI-2K: Systemic Lupus Erythematosus Disease Activity Index 2000
